# Potential Role of IL-17-Producing iNKT Cells in Type 1 Diabetes

**DOI:** 10.1371/journal.pone.0096151

**Published:** 2014-04-30

**Authors:** Shamin Li, Claudine Joseph, Chantal Becourt, Jihene Klibi, Sandrine Luce, Daniele Dubois-Laforgue, Etienne Larger, Christian Boitard, Kamel Benlagha

**Affiliations:** 1 Univ Paris Diderot, Sorbonne Paris Cité, Institut Universitaire d'Hématologie, Paris, France; 2 INSERM UMR1160, Paris, France; 3 Univ Paris Descartes, Sorbonne Paris Cité, Institut Cochin, Paris, France; 4 INSERM U1016, Paris, France; 5 Département de Bactériologie, Institut Pasteur, Paris, France; 6 Service de Diabétologie, Hôtel Dieu, GH Cochin-Hôtel Dieu-Broca, APHP et Univ Paris Descartes, Paris, France; Institut Pasteur, France

## Abstract

We explored in this study the status and potential role of IL-17-producing iNKT cells (iNKT17) in type 1 diabetes (T1D) by analyzing these cells in patients with T1D, and in NOD mice, a mouse model for T1D. Our analysis in mice showed an increase of iNKT17 cells in NOD *vs* control C57BL/6 mice, partly due to a better survival of these cells in the periphery. We also found a higher frequency of these cells in autoimmune-targeted organs with the occurrence of diabetes, suggesting their implication in the disease development. In humans, though absent in fresh PMBCs, iNKT17 cells are detected *in vitro* with a higher frequency in T1D patients compared to control subjects in the presence of the proinflammatory cytokine IL-1β, known to contribute to diabetes occurrence. These IL-1β-stimulated iNKT cells from T1D patients keep their potential to produce IFN-γ, a cytokine that drives islet β-cell destruction, but not IL-4, with a reverse picture observed in healthy volunteers. On the whole, our results argue in favour of a potential role of IL-17-producing iNKT cells in T1D and suggest that inflammation in T1D patients could induce a Th1/Th17 cytokine secretion profile in iNKT cells promoting disease development.

## Introduction

Invariant natural killer T (iNKT) cells constitute a peculiar population of T cells sharing phenotypical and functional characteristics of natural killer (NK) and T lymphocytes [1]. They are thymic-derived lymphocytes expressing a semi invariant T cell receptor (TCR) made of a Vα14-Jα18 rearranged α-chain in mice, paired with a limited set of Vβ chain (Vβ8,Vβ7 or Vβ2). Human iNKT cells express a TCR made of a unique Vα24-Jα18 chain associated with the Vβ11 chain. These cells express surface receptors belonging to the NK lineage such as NK1.1 in mice (CD161 in humans) and activating or inhibiting receptor (NKG2D or Ly-49) [1].

Both human and murine iNKT cells differ from conventional T cells as the TCR recognizes self and foreign lipid antigens presented by the non-polymorphic MHC class I-like antigen presenting molecule CD1d, present on macrophages, dendritic, and B cells [2].

The most studied glycolipid antigen recognized by iNKT cells is α-galactosylceramide (α-GalCer) that was initially extracted from a marine sponge. This antigen activates iNKT cells in mice and humans and its stable association with soluble CD1d allowed the generation of α-GalCer CD1d-tetramers (tet) that constitutes a powerful tool to track iNKT cells based on their TCR specificity [3].

Upon TCR activation, conventional iNKT cells rapidly produce high amounts of Th1 and Th2 cytokines namely IFN-γ and IL-4 respectively, that induce the activation of various immune cells including macrophages, NK, T and B cells [4]. Recently, several groups have reported a new subset of unconventional iNKT cells with the ability to secrete pro inflammatory Th17-related cytokines including interleukin-17 (IL-17) [5].

Diabetes is a tissue-specific autoimmune pathology affecting the pancreas, conducting to progressive destruction of insulin-producing beta-cells by autoreactive T lymphocytes infiltrate. Salivary glands are also prone to auto-destruction leading to sialitis [6].

Murine models and diabetic patients show lower frequencies of iNKT cells secreting IL-4 than non-diabetic subjects, suggesting a likely regulatory role of these cells in the development of the disease [7], [8]. Studies performed on the non-obese diabetic (NOD) mouse model, that develops spontaneous autoimmune diabetes as a result of Th1 mediated destruction of pancreatic islet cells, have revealed that activation of iNKT cells by α-GalCer ligand or thymic iNKT cell transfer prevents the onset of T1D [9]. Further confirmation on the protective role of iNKT cells was obtained using the Vα14 transgenic mouse model expressing high level of iNKT cells and large quantities of IL-4 [10]. Contrariwise, deficiency of iNKT cells in NOD mice has been proved to exacerbate the disease [11]. On the whole, these findings indicate an immunosuppressive role of conventional iNKT cells against anti-islet autoreactive T cells.

In humans, few reports exist concerning a role of conventional iNKT cells in T1D. The proposed link between iNKT cells and T1D rests on studies of discordant twin pairs, which reported a decreased frequency of iNKT cells in the peripheral blood of the diseased twin, as well as a complete and selective loss of the ability of iNKT cell clones to secrete IL-4 [7]. While these findings were supported by two studies [12], [13], it was contradicted by some having found an equal or higher frequency of Vα24-Vβ11 iNKT cells in the peripheral blood of diabetic patients [14], [15].

During these last years, our group and others have published several articles regarding the characterisation and mechanisms of action of the new iNKT17 cell subset in health and in response to bacteria [5], [16], [17]; however few studies analysed their role in autoimmunity. These iNKT17 cells differ from conventional iNKT cells in that they are NK1.1 negative in mice and express markers related to the Th17 lineage, such as IL-1 receptor, CCR6 and the retinoic acid receptor-related orphan receptor γt (RORγt); thus constituting a distinct non-conventional population of iNKT cells so-called RORγt^+^ iNKT or iNKT17 cells [5]. They are thought to belong to a divergent lineage that develops in the thymus and further migrates to localise mainly in peripheral lymph nodes (PLNs) where they mediate early innate-like immune responses [16].

As IL-17 response has been linked to various autoimmune diseases, we explored the contribution of iNKT17 in T1D. Our results show that iNKT17 cells are altered in NOD mice and T1D patients, and we propose, based on cytokine production analysis, that a modified cytokine balance of iNKT cells under inflammatory condition in T1D patients contributes to the development of the disease.

## Results

### Increased iNKT17 cell population in the periphery of NOD mice compared to C57BL/6 mice

**Figure 1:**
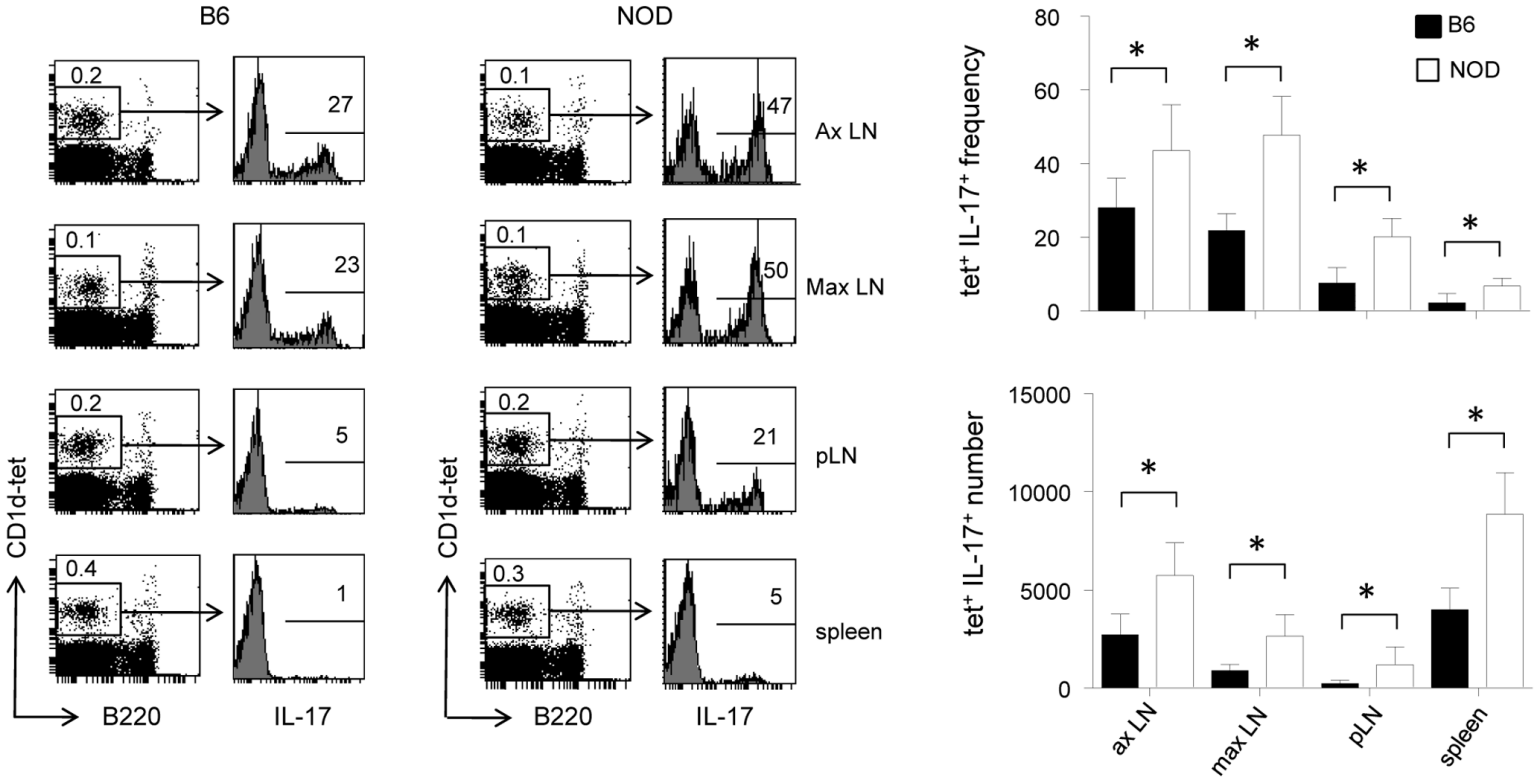
iNKT17 cells are increased in NOD mice compared with C57BL/6 mice. Axillary (Ax), maxillary (Max), and pancreatic (p) lymph nodes (LNs), and spleen cells from 12-wk-old C57BL/6 or NOD female mice were stimulated with PMA/Ionomycin in the presence of BFA for 4 hours. Cells were then stained with CD1d-tetramers (tet) and antibodies directed against B220 and IL-17A (IL-17). Representative dot and histogram plots are shown and numbers represent percentages. The frequency (upper histograms) and the absolute number (lower histograms) of IL-17^+^ cells among tet^+^ cells are shown. Data are presented as mean ± SD and are from 5 experiments where 3 to 4 mice per group were used in each experiment. *p<0.05, using non-parametric Mann-Whitney U test to determine significance.

To investigate the role of iNKT17 cells in T1D, we compared the frequency and absolute number of these cells in 12-wk-old NOD and C57BL/6 mice. C57BL/6 mice were used as control because they neither develop diabetes nor other autoimmune diseases. iNKT17 cells were analyzed in the spleen, axillary, maxillary, and pancreatic lymph nodes (LNs). Axillary LNs were used as control and are representative of peripheral lymph nodes (PLNs) since we showed in previous studies that PLNs are enriched in iNKT17 cells [5]. Maxillary and pancreatic LNs were used because they drain the salivary glands and the pancreas respectively and both organs are prone to progressive autoimmune destruction in NOD mice. IL-17 production by iNKT cells was detected after stimulation with phorbolmyrisistyl acetate (PMA) and ionomycin and CD1d-α-GalCer tetramer (tet) staining. As shown in figure 1, we found an increased frequency of tet^+^ IL-17-producing cells (tet^+^IL-17^+^) in NOD mice compared to C57BL/6 mice in all organs tested. This difference was also observed in terms of absolute number (Fig. 1). Because the expression of the transcription factor RORγt defines IL-17-producing cells, we wanted to confirm our results by comparing RORγt expression in tet^+^ cells in both strains of mice. We found that tet^+^RORγt^+^ cells have an increased frequency and absolute number in NOD mice compared to C57BL/6 mice (Fig. S1).

Analysis of congenic mice expressing NOD-related MHC H-2^g7^ on C57BL/6 background did not show an increase in RORγt^+^ iNKT cells (data not shown) indicating that additional NOD genes control increased RORγt^+^ iNKT cell numbers. Analysis of nonobese resistant (NOR) mice, in which H-2^g7^ is expressed on a genetic background 88% to NOD mice, would allow understanding if other genes non involved in disease development control RORγt^+^ iNKT cell number and if increased RORγt^+^ iNKT cell number could occur without diabetes. With this regard, our analysis of BALB/c mice, that do not develop diabetes, show an increased thymic RORγt^+^ iNKT cell population (data not shown) indicating that increased RORγt^+^ iNKT cells is not exclusive to NOD mice and thus not correlated to diabetes.

### Increased iNKT17 cell population in the thymus and periphery of young NOD mice

**Figure 2:**
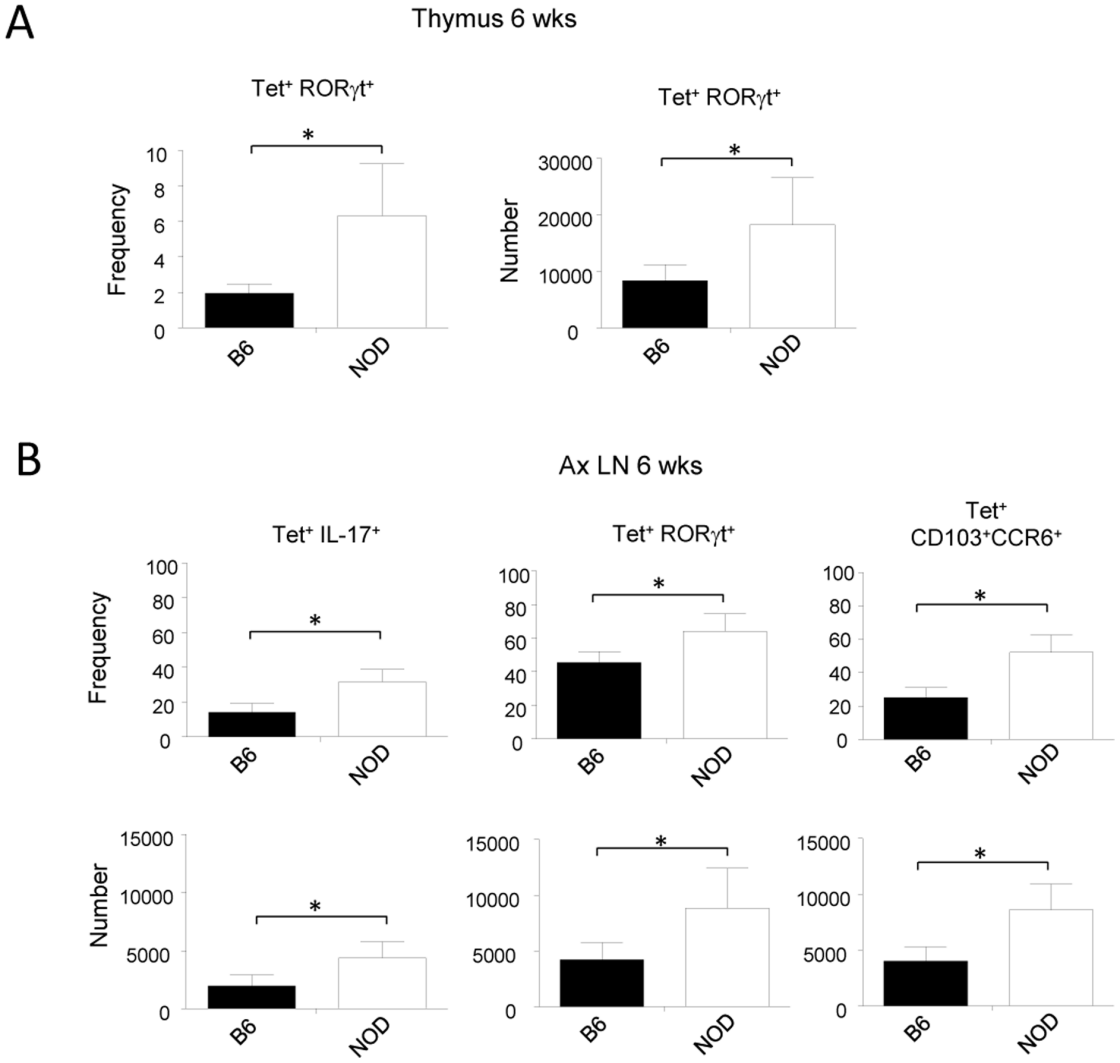
Increased iNKT17 cell population in the thymus and periphery of young NOD mice. **A**, Thymic cells from 6-wk-old C57BL/6 or NOD female mice were stained with CD1d- tetramers (tet) and then subjected to intracellular staining using anti-RORγt antibodies. The frequency and absolute number of RORγt^+^ cells among tet^+^ cells are shown. Data are presented as mean ± SD and are from 4 experiments where 3 to 4 mice per group were used in each experiment. **B**, Axillary LN cells (representative of maxillary, pancreatic LN and spleen) from 6-wk-old C57BL/6 or NOD female mice were subjected to surface or intracellular staining. The frequency and absolute number of IL-17^+^, RORγt^+^, and CD103^+^CCR6^+^ cells among tet^+^ cells are shown. Data are presented as mean ± SD and are from 4 experiments where 3 to 4 mice per group were used in each experiment. *p<0.05, using non-parametric Mann-Whitney U test to determine significance.

To determine if the increase of iNKT17 cells in NOD mice occurs at early ages before diabetes and in the thymus as well, we compared the frequency and absolute number of these cells in the thymus of 6-wk-old NOD and C57BL/6 mice. As shown in figure 2A, we found an increased frequency and absolute number of thymic tet^+^RORγt^+^ cells in NOD mice compared to C57BL/6 mice. An increase of IL-17-producing iNKT cells was also observed in NOD mice (data not shown). In these mice, we also observed an increase of iNKT17 cells in the periphery as exemplified by an increase in the frequency and absolute number of tet^+^IL-17^+^ and tet^+^RORγt^+^ cells in axillary LNs (Fig. 2B). The same results were observed in the spleen, maxillary, and pancreatic LNs (data not shown). We also observed an increase of tet^+^ cells co-expressing CCR6 and CD103, shown in previous studies to define RORγt^+^ IL-17-producing iNKT cells [5], in NOD mice compared to C57BL/6 mice (Fig. 2B). Our results thus indicate that the increased iNKT17 cell population in NOD mice resembles phenotypically the one observed in C57BL/6 mice. We also performed a functional and phenotypical comparative analysis using 4-wk-old mice and already observed an increase in the iNKT17 cell population (data not shown). Overall, these results indicate that the increase in iNKT17 cell population observed in NOD mice occurs very early in the thymus as well as in peripheral organs, before the onset of diabetes.

### Better survival of peripheral iNKT17 cells in NOD mice

**Figure 3:**
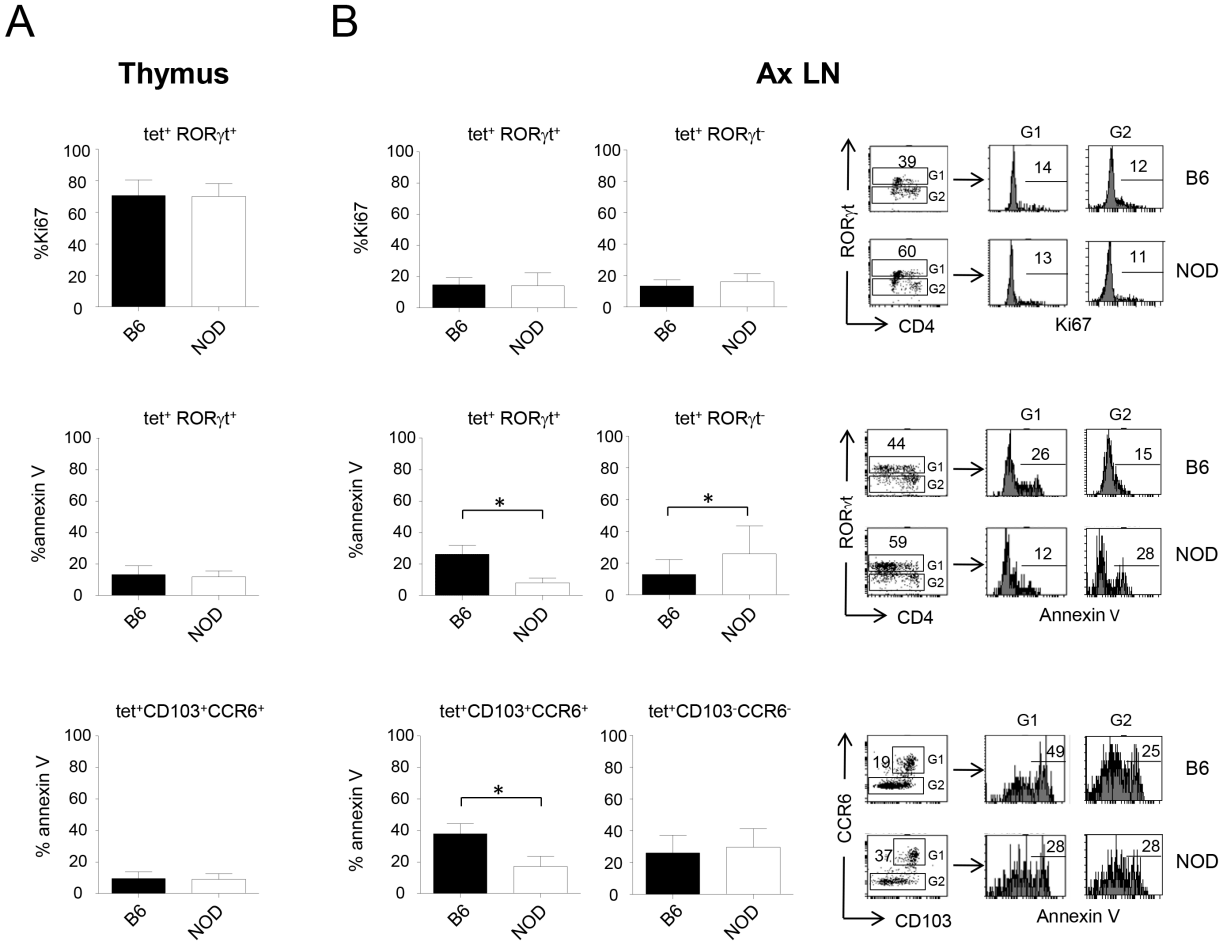
iNKT17 cells show a better survival in the periphery of NOD mice. Thymic (**A**) and axillary LN cells (**B**) from 6-wk-old C57BL/6 or NOD female mice were surface stained with CD1d-tetramers (tet) and Annexin V and subjected to intracellular staining with anti-RORγt and anti-Ki67 antibodies. Shown is the frequency of Ki67^+^ (upper panel) or annexin V^+^ cells (middle panel) among tet^+^RORγt^+^ iNKT cells and annexin V^+^ cells among tet^+^ CCR6^+^CD103^+^ iNKT cells (lower panel). Representative dot and histogram plots are shown for axillary LN and numbers represent percentages. Data are presented as mean ± SD and are from 4 experiments where 3 to 6 mice per group were used in each experiment. *p<0.05, using non-parametric Mann-Whitney U test to determine significance.

We next decided to investigate the origin of the increased iNKT17 cell population in NOD mice. To determine if this increase was due to a proliferative advantage, we compared the expression of Ki67, a nuclear cell proliferation-associated-Ag expressed in all active stages of the cell cycle, in thymic and peripheral iNKT17 cells. We found no difference in the expression of Ki67 in thymic tet^+^RORγt^+^ cells from NOD and C57BL/6 mice (Fig. 3A, upper panel). Also, no difference in Ki67 expression was observed when we compared axillary LNs from both strains of mice (Fig. 3B, upper panel). This was also the case when spleen, maxillary, or pancreatic LNs were tested (data not shown). This indicates that the increase of iNKT17 cells in NOD mice is not due to a proliferative advantage of these cells. We next tested the expression of annexin V, a phosphatidylserine expressed on apoptotic cells, on iNKT17 cells to assess whether the increased number of iNKT17 cells could be related to a better survival in the NOD mice. As shown in figure 3A (middle and lower panels), the frequency of annexin V^+^ cells among thymic tet^+^RORγt^+^ or tet^+^CCR6^+^CD103^+^ cells was comparable in NOD mice and in C57BL/6 mice. The frequency of annexin V^+^ cells among axillary LN iNKT17 cells was however lower in the NOD mice (Fig. 3B, middle and lower panels). The same results were observed for spleen, maxillary, and pancreatic LNs (data not shown). We also analyzed the frequency of annexin V^+^ cells in non-iNKT17 iNKT cells (Fig. 3B, middle and lower panels) and found that they do not benefit from the protection from apoptosis, as iNKT17 cells do in NOD mice, leading to a greater frequency of iNKT17 cells observed in NOD mice compared to C57BL/6 mice. Overall, these results show a better survival of iNKT17 cells in the periphery of NOD mice that could explain in part their increased number, in addition to a higher thymic production, and a greater export to the periphery.

### iNKT17 cells infiltrating the pancreas and salivary glands of NOD mice are increased in diabetic mice

**Figure 4:**
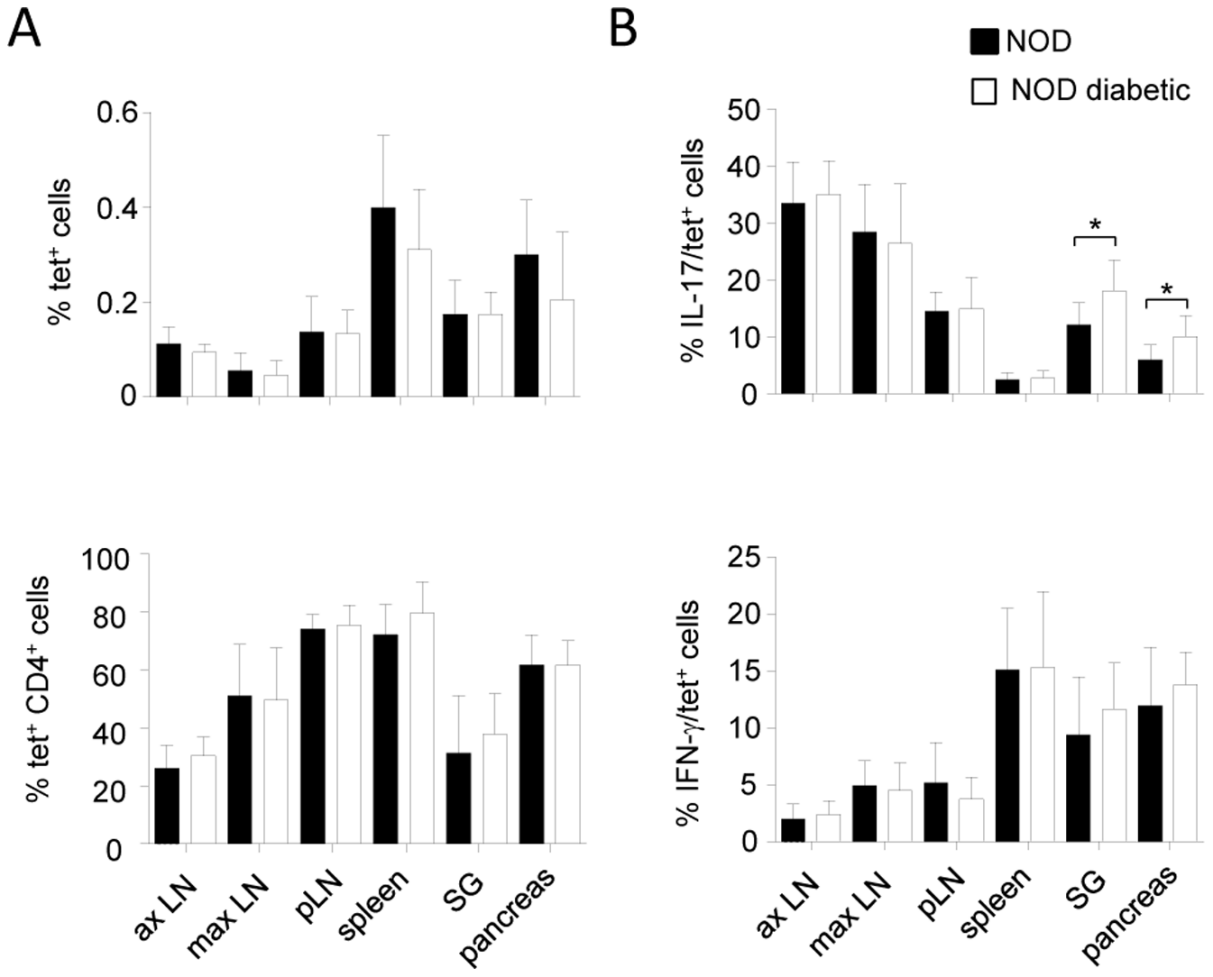
Increased iNKT17 cells in the pancreas and salivary glands of diabetic NOD mice. Frequency of tet^+^ and tet^+^CD4^+^ cells (**A**) and of IL-17^+^ and IFN-γ^+^ cells among tet^+^ cells (**B**) is assessed (by surface and intracellular staining as in figure 1 **)** in cells obtained from axillary (ax), maxillary (max), and pancreatic (p) LNs, spleen, pancreas, and salivary glands (SG) of same aged diabetic or non diabetic female NOD mice (20- to 22-wk-old). Data are presented as mean ± SD and are from 4 experiments where 4 to 5 mice per group were used in each experiment. *p<0.05, using non-parametric Mann-Whitney U test to determine significance.

To determine whether iNKT17 cells status varies with the occurrence of diabetes, we analysed iNKT cells in age-matched diabetic and non-diabetic female NOD mice. We found similar frequencies of tet^+^ or tet^+^CD4^+^ iNKT cells in diabetic NOD mice compared to non-diabetic ones (Fig. 4A, and S2 for representative dot plots). Interestingly, we found a slightly higher frequency of tet^+^IL-17^+^ cells in the pancreas and salivary glands of diabetic NOD mice (Fig. 4B, upper panel) but not in the pancreatic and maxillary LNs, draining the pancreas and salivary glands, respectively. Also, no difference was observed in the spleen and axillary LNs. The increase in the frequency of iNKT17 cells in the pancreas and salivary glands was confirmed by the analysis of tet^+^RORγt^+^ cells (data not shown). Interestingly, we did not observe any increase in the frequency of tet^+^IFN-γ^+^ cells in the pancreas and salivary glands or in any other organ tested in the diabetic NOD mice (Fig. 4B, lower panel, and S2 for representative dot plots). We also compared the frequency of CD4^+^, CD8^+^/NK^+^, and γδ^+^ cells and their capacity to produce IL-17 and IFN-γ and no difference was detected (Fig. S2). Overall, these results show an increase in the frequency of iNKT17 cells in the target organs of the autoimmune process in diabetic mice, suggesting that these cells might be involved in the development of the disease.

### iNKT17 cells are not detectable in T1D patients

**Figure 5:**
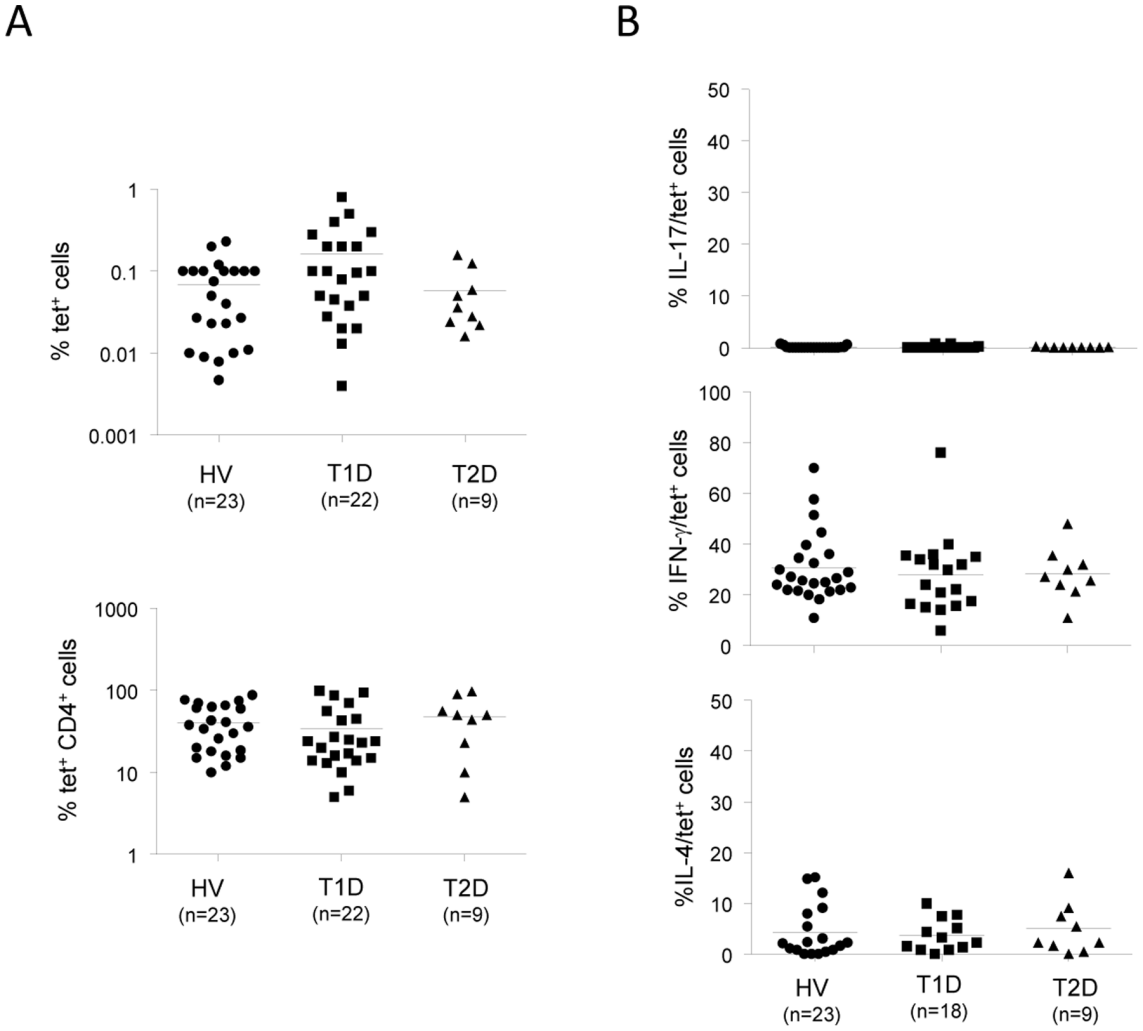
iNKT17 cells are not detected in patients with type 1 diabetes. Fresh human PBMCs are stained with CD1d-tetramers (tet), anti-CD3, and anti-CD4 mAbs, and, in parallel, stimulated with PMA/Ionomycin. Four hours later, cells were subjected to intracellular staining to assess IL-17, IFN-γ, or IL-4 production. **A**, Shown is the frequency of tet^+^ cells and tet^+^CD4^+^ cells observed in patients with type 1 diabetes (T1D), control Healthy volunteers (HV), or patients with type 2 diabetes (T2D). **B**, Shown is the frequency of IL-17^+^, IFN-γ^+^, and IL-4^+^ cells among tet^+^ cells in T1D patients, HV and T2D patients. Numbers represent percentages. Each symbol represents one individual and horizontal bars indicate mean ± SD. n: number of subjects tested.

It has been shown in one study that iNKT17 cells are not detected in human PBMCs [18]. Based on our results in the NOD mouse model showing increased iNKT17 cells in the pancreas and salivary glands of diabetic mice, we analysed the frequency of these cells in patients with T1D, assuming that they might be present in an autoimmune condition such as T1D. iNKT cells were detected using CD1d-α-GalCer tetramers (tet) and no difference in their frequency, or their CD4 expression, was observed when compared to control groups (healthy volunteers and T2D patients) (Fig. 5A and S3A for representative dot plots). We found that IL-17^+^ cells among tet^+^ cells, determined after PMA/Ionomycin stimulation, are absent (Fig. 5B, upper panel, and S3A for representative dot plots). IL-17^+^ cells are also absent in control HV and T2D patients. These results were confirmed using RORγt as a surrogate marker to identify iNKT17 cells (data not shown). On the contrary, iNKT cells from HV, T1D, and T2D patients comprise IFN-γ and IL-4 producing cells and no difference in cytokine production was observed between the three groups (Fig. 5B, middle and lower panels). We also compared cytokine production (IL-17, IFN-γ, and IL-4) by CD3^+^cells between the three groups and found no difference (Fig. S3B). Nearly half of T1D patients were tested twice or three times over a period of at least one year and there was no significant difference in tet^+^ cell frequencies and cytokine production capabilities, indicating the reproducibility of our assay and confirming previous studies showing their stability over time in a given individual [14].

### iNKT cells expand *in vitro* in the presence of the Th17-related cytokine IL-1

We reasoned that iNKT17 cells are rare and that we might be able to detect them after expansion *in vitro* or after conversion from iNKT cells in the presence of appropriate inflammatory cytokines. Human iNKT cells from healthy volunteers were shown to expand in the presence of α-GalCer and IL-2, and IL-7 or IL-15 could substitute for IL-2 or potentiate its response [19], [20], [21]. We thus tested whether IL-7 or IL-15 could promote a proliferative response of iNKT cells from diabetic patients in the presence of α-GalCer and IL-2. We found that IL-7 and IL-15 induced the proliferation of iNKT cells from diabetic patients as assessed by tet staining after 10 days of culture (Fig. S4A). The combination of both cytokines did not increase this response (data not shown). Analysis of the fold increase in iNKT cell number (after 10 days of culture/at the start of the culture) indicated that iNKT cells from diabetic patients and healthy volunteers responded equally to IL-7 and IL-15 (Fig. S4B). Because our ultimate goal through these amplification assays was to unravel iNKT17 cells, we tested the effect of the Th17-related cytokines IL-1β and IL-23, in the presence of IL-2, on the proliferation capabilities of iNKT cells. Interestingly, we found that IL-1β, alone, induces the best proliferation of iNKT cell when compared to the conventional cytokines IL-7 and IL-15 (Fig. S4B). Paired analysis indicated that IL-1 induced in most cases a stronger proliferation than the other cytokines tested (Fig. S4C). These results were observed for iNKT cells from healthy volunteers and diabetic patients as well (Fig. S4C). Comparison of the fold increase in cell number between HV and T1D patients indicated that iNKT cells from both groups respond equally well to IL-1β and IL-23 (Fig. S4B). Overall, these results show that iNKT cells from T1D patients are able to expand *in vitro*. This expansion occurs not only in the presence of the homeostatic cytokines IL-7 and IL-15, but also in the presence of pro-inflammatory cytokines, mostly IL-1β.

### IL-1-expanded iNKT cells produce IL-17 and have a higher frequency in T1D patients

**Figure 6:**
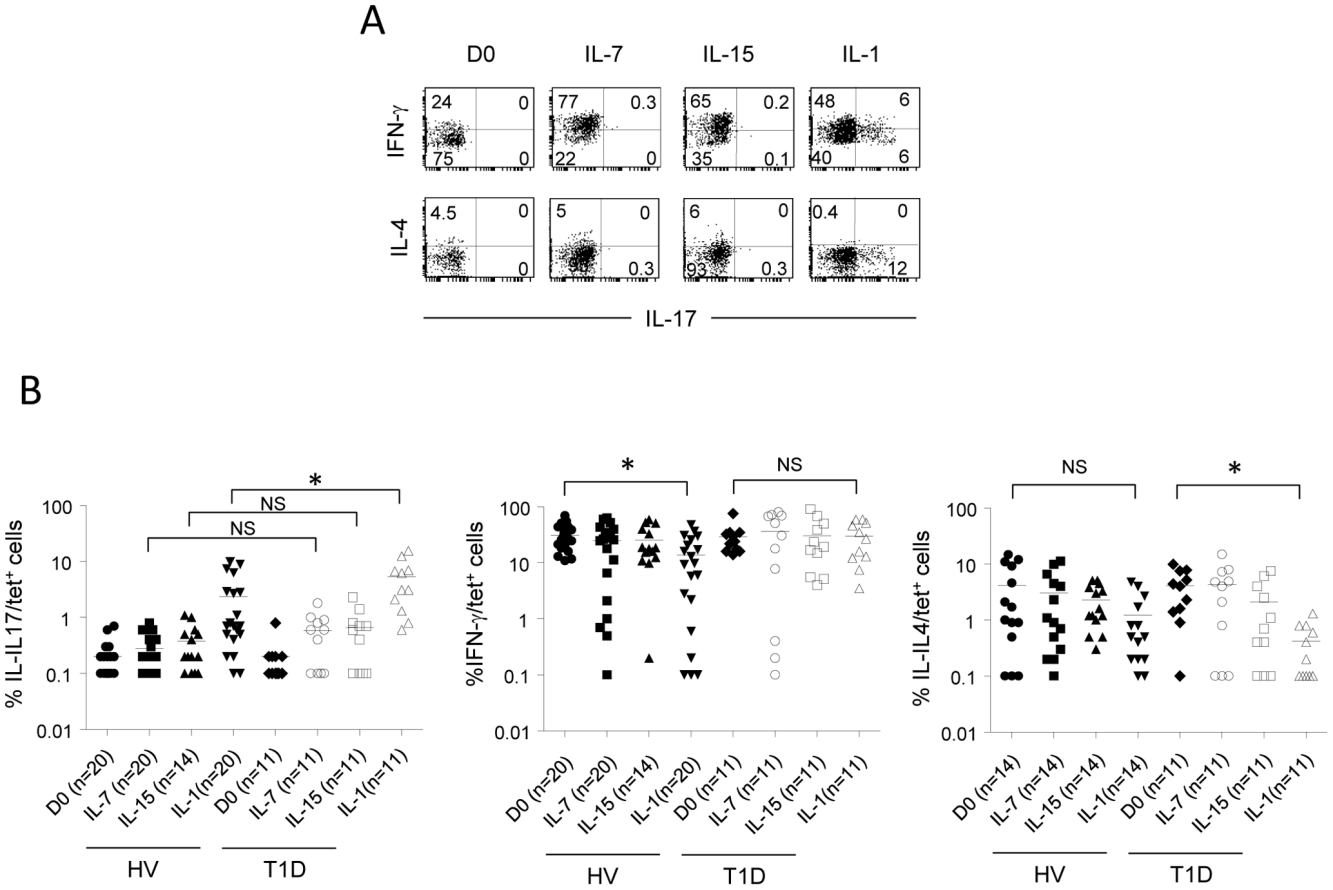
IL-17-producing iNKT cells are detected after expansion of iNKT cells in presence of proinflammatory cytokines. **A**, PBMCs from healthy volunteers (HV) and patients with type 1 diabetes (T1D) were cultured for 10 days as in figure S4 in the presence of IL-2, α-GalCer, and additional cytokines (IL-7, IL-15, or IL-1β). After 10 days of culture, expanded cells were stimulated with PMA and Ionomycin in the presence of BFA and the expression of IL-17, IFN-γ, and IL-4 was evaluated after intracellular staining. Shown are representative dot plots obtained from a T1D patient of IL-17 detection along with IFN-γ and IL-4. Numbers represent percentages. **B**, The frequency of IL-17^+^, IFN-γ^+^, and IL-4^+^ cells among tet^+^ cells for each subject is shown. Each symbol represents one individual and horizontal bars indicate mean. NS: not significant. n: number of subject tested. *p<0.05, using non-parametric Mann-Whitney U test to determine significance.

To evaluate IL-17 production by expanded iNKT cells, stimulated cells with PMA/Ionomycin were subjected to intracellular staining to identify IL-17 producers among tet^+^ cells. We found that only IL-1β-expanded iNKT cells produce IL-17 (Fig. 6A for representative dot plots, and 6B, left panel). A previous report indicated that human iNKT cells require the concomitant presence of TGF-β, IL-1β and IL-23 to produce IL-17 [18]. Our results show that IL-1β alone is sufficient, although we do not exclude the presence of TGF-β in culture media. Importantly, we found that expanded iNKT cells from T1D patients comprise a higher frequency of iNKT17 cells compared to HV (Fig. 6B, left panel). These increased iNKT17 cells are not likely to be connected to glucose homeostasis or insulin treatment, as it is not observed in T2D patients (data not shown), but likely to the inflammation associated with T1D. This result suggests that even though iNKT17 cells are not detected in human PBMCs, they can be functionally induced under pro-inflammatory environment. Additionally, we found that some IL-1β-induced iNKT17 cells co-produce IFN-γ, arguing in favour of conversion of iNKT cells secreting IFN-γ into iNKT17 cells and suggesting that iNKT cells from T1D patients are likely to be more prone to be converted to IL-17-producing cells in the presence of appropriate inflammatoty cytokines. Furthermore, IL-1β-expanded iNKT cells from T1D patients keep their capacity to produce IFN-γ but not IL-4 (Fig. 6B, middle and right panels). This is contrary to what observed for HV where iNKT cells keep their ability to produce IL-4 but not IFN-γ (Fig. 6B, middle and right panels). This outcome indicates that IL-1β favours a Th1/Th17 cytokine secretion profile of iNKT cells in T1D patients.

Overall, our results show an altered cytokine balance of iNKT cells under inflammatory condition in T1D patients that could contribute to the development of this disease.

## Discussion

In this present study, we analysed iNKT17 cells in autoimmune NOD mice and in patients with T1D and found that iNKT17 cells are increased in the pancreas and salivary glands of diabetic NOD mice and in patients with T1D, suggesting that these cells could be involved in the development of the disease.

Our study is in accordance with a study by Simoni et al. showing an increase of IL-17-secreting iNKT cells in the thymus, spleen, inguinal and peripheral LNs of NOD mice as compared with C57BL/6 mice following stimulation with PMA/Ionomycin [22]. Furthermore, our study showed that this increase of iNKT17 cells is not the result of higher proliferation but could be due to an increased survival in the periphery that might explain in part their increased number in NOD mice. It has been shown that autoimmune inflammation in NOD mice begins at around 4 weeks of age with peri-insulitis and infiltration of macrophages and dendritic cells; and monocytes from NOD mice and T1D patients have been reported to spontaneously secrete pro-inflammatory cytokines that drive Th17 cells [23], [24], [25]. Our own studies have shown that iNKT17 cells respond to the pro-inflammatory cytokines IL-1 and IL-23 in the peripheral lymph nodes of B6 mice [16]. We could thus hypothesize that the increased survival of iNKT17 cells in NOD mice is the result of strain-related pro-inflammatory environment.

Analysis of the frequency of iNKT17 cells between diabetic and non-diabetic NOD mice indicates a higher frequency of these cells in the pancreas of diabetic ones, which is a target of autoimmune destruction indicating that these iNKT17 cells might be involved in the pathogenesis of T1D. These cells are unlikely to be involved in the initiation of the disease given the fact that diabetes has been reported in iNKT cell deficient CD1d KO mice [11], although they may be implicated in disease severity. The implication of these cells in the development of the disease has been shown in a study by Simoni et al. through transfer studies where co-transferring NK1.1^−^ CD4^−^ iNKT cells, mainly containing iNKT17 cells and obtained from Vα14 transgenic NOD mice, with diabetogenic BDC2.5 T cells into NOD/SCID (severe combined immunodeficiency NOD) mouse model exacerbates T1D [22]. IL-17 produced by transferred cells is likely to be at the origin of the pathogenic effect of transferred cells since the presence of anti-IL-17 antibodies abrogated this pathogenic effect [22].

Another argument in favour of the importance of IL-17 in diabetes are studies from Emamaullee et al. who reported that anti-IL-17 treatment of 10-wk-old NOD mice reduced diabetes frequency [26]. *In vitro* data showed that IL-17 can synergise with cytokines such as IL-1β and IFN-γ to induce iNOS expression and subsequent NO production in pancreatic islets of NOD mice [27]. Thus, IL-17 production by iNKT17 cells observed in the pancreas of diabetic NOD mice may contribute to the occurrence of T1D by synergizing with other locally produced cytokines, such as IFN-γ, to induce high expression of NO in β-cells resulting in their destruction.

Even though our findings suggest a role of iNKT17 cells in the pathogenesis of T1D, IL-17 is also secreted by other immune cells such as Th17 and γδ T cells. The role of Th17 cells in T1D remains imprecise. In fact, the induction of the disease in NOD SCID mice after transfer of *in vitro* polarized Th17 cells anti-islet T cells was abolished by anti-IL-17 treatment in one study but not in two others [28], [29], [30]. Regarding γδ T cells, it has been shown that IL-17-secreting γδ T cells have no effect in the incidence of T1D upon co-transfer into NOD SCID mice [31]. Another study by Markle et al. showed a pathogenic role of IL-17 produced by γδ T cells [32]. In our study, we did not observe a difference in the frequency of IL-17-producing αβ CD4^+^ and γδ T cells when comparing the pancreas and the salivary glands of diabetic and non-diabetic female NOD mice.

We also analysed iNKT17 cells in human T1D patients and found a higher frequency of these cells in T1D patients compared to HV when expanded in presence of IL-1β. The origin of the increased frequency of iNKT17 cells in T1D patients is unknown, but it has been shown that patients with T1D have increased monocytic activity and biomarkers of inflammation including IL-1β [25]. In view of that, inflammation associated with T1D might be at the origin of iNKT17 cells increase. Along with this line, monocytes isolated from patients with T1D spontaneously secrete the pro-inflammatory cytokines IL-1 and IL-6, which are known to induce and expand Th17 cells [24]. Moreover, these *in vivo-*activated monocytes induce more IL-17-secreting cells from memory T cells compared with monocytes from healthy control subjects [24]. Importantly, it was reported that the frequency of IL-17-secreting CD4^+^ T cells in lymphocytes from established T1D patients was increased compared to healthy controls [22]. No increase in IL-17-secreting CD4^+^ T cells in new-onset T1D patients was observed in this study. However, the relevance of IL-17-secreting CD4^+^ T cells in new-onset T1D patients was shown in three subsequent studies [33], [34], [35]. In one of these studies, IL-17-secreting CD4^+^ T cells specific for β-cell antigens are present in the circulation and IL-17A transcripts are elevated in the pancreatic islets near to diagnosis of T1D [33].

Besides, we also found that IL-1β-induced iNKT17 cells maintain their capacity to secrete IFN-γ which is the main cytokine that drives islet β-cell destruction. We could thus hypothesise that IL-17 and IFN-γ synergy to cause exacerbation of pancreatic β-cells destruction in T1D. It is important to note that IL-17 receptor up-regulation by the pro-inflammatory cytokines IL-1β and IFN-γ has been reported to make human β-cells highly susceptible to death through apoptosis by combination of IL-17, IL-1β, and IFN-γ [30]. Along this line, a recent study has demonstrated that double deficiency of IL-17 and IFN-γ signaling suppresses the development of diabetes in the NOD mouse [36].

Altogether, our study shows that iNKT17 cells could be implicated in the pathogenesis of T1D and suggests that an altered cytokine balance of iNKT cells under inflammatory condition in T1D patients could contribute to the development of this disease. Further studies are needed to better understand their precise function in T1D, and their implication in other autoimmune diseases.

## Materials and Methods

### Ethics statement

Experimental studies were in accordance with the Hôpital Cochin St Vincent de Paul Institutional Animal Care and Use Guidelines. All experimental protocols in this study were approved by the University Paris V, Descartes, ethics committee. All efforts were made to minimize mice suffering.

Hôpital Cochin St Vincent de Paul ethical committee approval was received for the human studies and informed written consent was obtained from all patients.

### Mice

C57BL/6 mice were purchased from Janvier. NOD mice were purchased from The Jackson Laboratory. All mice were maintained under specific pathogen-free conditions at the Hôpital Cochin St Vincent de Paul animal facility.

### Diabetes diagnosis

Mice were tested every day for diabetes. Overt diabetes was defined as two positive urine glucose tests, confirmed by a glycemia>300 mg/dl. Glukotest and Heamoglukotest kits were purchased from Roche.

### Patients

T1D was defined as positivity for islet cell antibodies, anti-GAD, anti-insulin, or anti-IA2 antibodies at diabetes diagnosis. Long-standing T1D patients (n = 22, 14 males and 8 females, aged 44±10 years (range 25–63)), had been treated with insulin for 2–41 years at the time of study (mean T1D duration, 14±12 years). Control subjects were healthy blood donors (n = 23, 12 males and 11 females, aged 39±10 years (range 25–65), and type 2 diabetic patients (n = 9, 5 males and 4 females, aged 59±12 years (range 49–64)) on insulin treatment.

### Antibodies

Fluorochrome conjugates of antibodies against human Vβ11 (C21; Beckman Coulter), IFN-γ (4S.B3; Biolegend), CD4 (SK3; BD Biosciences), CD3 (HIT3a), CD8 (RPA-T8), IL-17 (64DEC17), and IL-4 (8D4-8), all from eBioscience, were used. Fluorochrome conjugates of antibodies against mouse CD24 (M1/69; Biolegend), B220 (RA3-6B2), CCR6 (140706), and IL-17 (TC11-18H10), all from BD Biosciences, CD4 (RM4-5), CD103 (2E7), Ki-67 (SolA15), IFN-γ (XMG1.2) and RORγt (AFKJS-9), all from eBioscience, were used.

### Extraction of pancreatic and salivary gland infiltrating lymphocytes

Each pancreas and salivary gland was directly and individually incubated in RPMI 1640 containing collagenase type VIII (Sigma-Aldrich) and Dnase I (Sigma-Aldrich), and cell suspensions were obtained as described previously [5].

### Flow cytometry

Cell suspensions were obtained from thymus, spleen, and lymph nodes (LNs) of C57BL/6 or NOD mice. PBMCs were obtained from whole blood by centrifugation over Ficoll (Amersham Pharmacia Biotech) gradient. Staining with CD1d-α-GalCer tetramers for mice or human cells was as follows. Cells were incubated with 1 µg/ml unlabeled streptavidin (Pierce Chemical Co.) for 15 min at room temperature, followed by incubation with CD1d-α-GalCer tetramers for 30 mn at 37°C. Other monoclonal antibodies (mAbs) were then added for further 30 min incubation on ice. Cells were then washed with staining buffer (PBS, 0.1% BSA; Sigma-Aldrich) and 0.01% sodium azide (Sigma-Aldrich) and analyzed by flow cytometry using FACSort and DIVA software (Becton Dickinson). For studies involving intracellular cytokines, cells were first stained with tetramers, then permeabilized with Fixation/Permeabilization solution (eBioscience), and washed with Permeabilization buffer (eBioscience). Appropriate mAbs were then added for 30 min before two further washes with permeabilization buffer. Annexin V Apoptosis Detection Kit with 7-AAD was used according to the manufacturer's instructions.

### Cell culture

Human donor PBMCs (2×10^6^/ml) were cultured in complete RPMI 1640 medium (GibcoBRL) and 10% FCS for 10 days in the presence of α-GalCer along with rhIL-2 (10 ng/ml) in addition to the following cytokines added individually or in combination: rhIL-7 (10 ng/ml), rhIL-15 (10 ng/ml), rhIL-1β (5 ng/ml), rhIL-23 (10 ng/ml) (all from PeproTech).

### Stimulation of cytokine production

Mice cells or PBMCs were cultured in the presence of 10ng/ml of phorbol-myristateacetate (PMA), 0.5 µg/ml of ionomycin, and 5 µg/ml of Brefeldin A (all from Sigma-Aldrich) for 4 hours before intracellular staining.

### Statistical analysis

Non-parametric Mann-Whitney U test for statistical analysis was used.

## Supporting Information

Figure S1 **RORγt^+^ iNKT cells are increased in NOD mice compared with C57BL/6 mice.** Axillary (ax), maxillary (max), and pancreatic (p) LNs, and spleen cells from 12-wk-old C57BL/6 or NOD female mice were stained with CD1d-tetramers (tet) and antibodies directed against the transcription factor RORγt. The frequency and the absolute number of RORγt^+^ cells among tet^+^ cells (left), and representative histogram plots (right) are shown. Numbers represent percentages. Data are presented as mean ± SD and are from 5 experiments where 3 to 4 mice per group were used in each experiment. *p<0.05, using non-parametric Mann-Whitney U test to determine significance.
(TIF)

Figure S2 **Unaltered cytokine production of CD4^+^, NK^+^/CD8^+^, and γδ^+^ cells of diabetic NOD mice.** **A,** Axillary (ax), maxillary (max), and pancreatic (p) LNs, and spleen cells from same aged diabetic or control non diabetic NOD female mice (20- to 22-wk-old) were stimulated with PMA/Ionomycin in the presence of BFA for 4 hours. Cells were then surface stained with CD1d-tetramers and antibodies directed against B220, CD4, and TCRγδ, followed by intracellular staining to detect IL-17A (IL-17) and IFN-γ. Shown are representative dot plots and gating strategies to determine cell population frequencies and cytokine production. Numbers represent percentages. **B**, Shown is the frequency of CD4^+^, CD8^+^/NK^+^, and γδ^+^ cells and the frequency of IL-17^+^ and IFN-γ^+^ cells among these cells. Data are presented as mean ± SD and are from 4 experiments where 4 to 5 mice per group were used in each experiment.
(TIF)

Figure S3 **Unaltered cytokine production of CD3^+^ cells in patients with type 1 diabetes.** **A**, Fresh human PBMCs were stained with CD1d-tetramers (tet) and anti-CD3 antibodies after being stimulated with PMA/Ionomycin. Cells were then subjected to intracellular staining to assess IL-17, IFN-γ, or IL-4 production. Shown are representative dot plots of tet-staining and IL-17, IFN-γ, and IL-4 production among tet^+^ or CD3^+^tet^−^ cells from a patient with type 1 diabetes (T1D). **B**, Shown is the frequency of IL-17^+^, IFN-γ^+^, and IL-4^+^ cells among CD3^+^tet^−^ cells in Healthy volunteers (HV), T1D patients, and patients with type 2 diabetes (T2D). Each symbol represents one individual and horizontal bars indicate mean ± SD. n: number of subjects tested.
(TIF)

Figure S4 **iNKT cells expand** ***in vitro*** **in the presence of the proinflammatory cytokine IL-1β.** **A,** Human PBMCs from healthy volunteers (HV) or type 1 diabetes (T1D) were cultured for 10 days in the presence of IL-2, α-GalCer and the mentioned cytokine. Proliferation of iNKT cells (gated as tet^+^CD3^+^ cells) is evaluated. Representative dot plots of CD1d tetramer staining from a T1D patient before (D0) and after expansion (D10) under the mentioned conditions are shown. Numbers represent percentages. **B**, Fold increase of iNKT cell number for each subject tested (after 10 days of culture/at the start of the culture) is shown. Each symbol represents one individual and horizontal bars with numbers indicate mean. NS: not significant. n: number of subjects tested. **C**, Shown are paired comparison between tet^+^ cell numbers observed after 10 days of culture with the mentioned cytokines.
(TIF)

## References

[1] Bendelac A, Savage PB, Teyton L (2007) The biology of NKT cells. Annu Rev Immunol 25: 297–336. 17150027 10.1146/annurev.immunol.25.022106.141711

[2] Brennan PJ, Brigl M, Brenner MB (2013) Invariant natural killer T cells: an innate activation scheme linked to diverse effector functions. Nat Rev Immunol 13: 101–117. 23334244 10.1038/nri3369

[3] Benlagha K, Weiss A, Beavis A, Teyton L, Bendelac A (2000) In vivo identification of glycolipid antigen-specific T cells using fluorescent CD1d tetramers. J Exp Med 191: 1895–1903. 10839805 10.1084/jem.191.11.1895 PMC2213523

[4] Chen H, Paul WE (1997) Cultured NK1.1+ CD4+ T cells produce large amounts of IL-4 and IFN-gamma upon activation by anti-CD3 or CD1. J Immunol 159: 2240–2249. 9278312

[5] Doisne JM, Becourt C, Amniai L, Duarte N, Le Luduec JB, et al (2009) Skin and peripheral lymph node invariant NKT cells are mainly retinoic acid receptor-related orphan receptor (gamma)t+ and respond preferentially under inflammatory conditions. J Immunol 183: 2142–2149. 19587013 10.4049/jimmunol.0901059

[6] Larger E, Becourt C, Bach JF, Boitard C (1995) Pancreatic islet beta cells drive T cell-immune responses in the nonobese diabetic mouse model. J Exp Med 181: 1635–1642. 7722443 10.1084/jem.181.5.1635 PMC2192008

[7] Wilson SB, Kent SC, Patton KT, Orban T, Jackson RA, et al (1998) Extreme Th1 bias of invariant Valpha24JalphaQ T cells in type 1 diabetes. Nature 391: 177–181. 9428763 10.1038/34419

[8] Gombert JM, Herbelin A, Tancrede-Bohin E, Dy M, Carnaud C, et al (1996) Early quantitative and functional deficiency of NK1+−like thymocytes in the NOD mouse. Eur J Immunol 26: 2989–2998. 8977295 10.1002/eji.1830261226

[9] Sharif S, Arreaza GA, Zucker P, Mi QS, Sondhi J, et al (2001) Activation of natural killer T cells by alpha-galactosylceramide treatment prevents the onset and recurrence of autoimmune Type 1 diabetes. Nat Med 7: 1057–1062. 11533711 10.1038/nm0901-1057

[10] Lehuen A, Lantz O, Beaudoin L, Laloux V, Carnaud C, et al (1998) Overexpression of natural killer T cells protects Valpha14- Jalpha281 transgenic nonobese diabetic mice against diabetes. J Exp Med 188: 1831–1839. 9815260 10.1084/jem.188.10.1831 PMC2212408

[11] Wang B, Geng YB, Wang CR (2001) CD1-restricted NK T cells protect nonobese diabetic mice from developing diabetes. J Exp Med 194: 313–320. 11489950 10.1084/jem.194.3.313 PMC2193465

[12] Kukreja A, Cost G, Marker J, Zhang C, Sun Z, et al (2002) Multiple immuno-regulatory defects in type-1 diabetes. J Clin Invest 109: 131–140. 11781358 10.1172/JCI13605 PMC150819

[13] Kis J, Engelmann P, Farkas K, Richman G, Eck S, et al (2007) Reduced CD4+ subset and Th1 bias of the human iNKT cells in Type 1 diabetes mellitus. J Leukoc Biol 81: 654–662. 17151140 10.1189/jlb.1106654

[14] Lee PT, Putnam A, Benlagha K, Teyton L, Gottlieb PA, et al (2002) Testing the NKT cell hypothesis of human IDDM pathogenesis. J Clin Invest 110: 793–800. 12235110 10.1172/JCI15832 PMC151131

[15] Oikawa Y, Shimada A, Yamada S, Motohashi Y, Nakagawa Y, et al (2002) High frequency of valpha24(+) vbeta11(+) T-cells observed in type 1 diabetes. Diabetes Care 25: 1818–1823. 12351484 10.2337/diacare.25.10.1818

[16] Doisne JM, Soulard V, Becourt C, Amniai L, Henrot P, et al (2011) Cutting edge: crucial role of IL-1 and IL-23 in the innate IL-17 response of peripheral lymph node NK1.1- invariant NKT cells to bacteria. J Immunol 186: 662–666. 21169541 10.4049/jimmunol.1002725

[17] Michel ML, Keller AC, Paget C, Fujio M, Trottein F, et al (2007) Identification of an IL-17-producing NK1.1(neg) iNKT cell population involved in airway neutrophilia. J Exp Med 204: 995–1001. 17470641 10.1084/jem.20061551 PMC2118594

[18] Moreira-Teixeira L, Resende M, Coffre M, Devergne O, Herbeuval JP, et al (2011) Proinflammatory environment dictates the IL-17-producing capacity of human invariant NKT cells. J Immunol 186: 5758–5765. 21478400 10.4049/jimmunol.1003043

[19] Fujii S, Shimizu K, Steinman RM, Dhodapkar MV (2003) Detection and activation of human Valpha24+ natural killer T cells using alpha-galactosyl ceramide-pulsed dendritic cells. J Immunol Methods 272: 147–159. 12505720 10.1016/s0022-1759(02)00497-0

[20] Lin H, Nieda M, Nicol AJ (2004) Differential proliferative response of NKT cell subpopulations to in vitro stimulation in presence of different cytokines. Eur J Immunol 34: 2664–2671. 15368282 10.1002/eji.200324834

[21] Rogers PR, Matsumoto A, Naidenko O, Kronenberg M, Mikayama T, et al (2004) Expansion of human Valpha24+ NKT cells by repeated stimulation with KRN7000. J Immunol Methods 285: 197–214. 14980434 10.1016/j.jim.2003.12.003

[22] Simoni Y, Gautron AS, Beaudoin L, Bui LC, Michel ML, et al (2011) NOD mice contain an elevated frequency of iNKT17 cells that exacerbate diabetes. Eur J Immunol 41: 3574–3585. 22002883 10.1002/eji.201141751

[23] Bertin-Maghit S, Pang D, O'Sullivan B, Best S, Duggan E, et al (2011) Interleukin-1beta produced in response to islet autoantigen presentation differentiates T-helper 17 cells at the expense of regulatory T-cells: Implications for the timing of tolerizing immunotherapy. Diabetes 60: 248–257. 20980463 10.2337/db10-0104 PMC3012178

[24] Bradshaw EM, Raddassi K, Elyaman W, Orban T, Gottlieb PA, et al (2009) Monocytes from patients with type 1 diabetes spontaneously secrete proinflammatory cytokines inducing Th17 cells. J Immunol 183: 4432–4439. 19748982 10.4049/jimmunol.0900576 PMC2770506

[25] Devaraj S, Glaser N, Griffen S, Wang-Polagruto J, Miguelino E, et al (2006) Increased monocytic activity and biomarkers of inflammation in patients with type 1 diabetes. Diabetes 55: 774–779. 16505242 10.2337/diabetes.55.03.06.db05-1417

[26] Emamaullee JA, Davis J, Merani S, Toso C, Elliott JF, et al (2009) Inhibition of Th17 cells regulates autoimmune diabetes in NOD mice. Diabetes 58: 1302–1311. 19289457 10.2337/db08-1113 PMC2682686

[27] Miljkovic D, Cvetkovic I, Momcilovic M, Maksimovic-Ivanic D, Stosic-Grujicic S, et al (2005) Interleukin-17 stimulates inducible nitric oxide synthase-dependent toxicity in mouse beta cells. Cell Mol Life Sci 62: 2658–2668. 16261264 10.1007/s00018-005-5259-0 PMC11139109

[28] Jain R, Tartar DM, Gregg RK, Divekar RD, Bell JJ, et al (2008) Innocuous IFNgamma induced by adjuvant-free antigen restores normoglycemia in NOD mice through inhibition of IL-17 production. J Exp Med 205: 207–218. 18195074 10.1084/jem.20071878 PMC2234380

[29] Bending D, De la Pena H, Veldhoen M, Phillips JM, Uyttenhove C, et al (2009) Highly purified Th17 cells from BDC2.5NOD mice convert into Th1-like cells in NOD/SCID recipient mice. J Clin Invest 119: 565–572. 19188681 10.1172/JCI37865 PMC2648686

[30] Martin-Orozco N, Chung Y, Chang SH, Wang YH, Dong C (2009) Th17 cells promote pancreatic inflammation but only induce diabetes efficiently in lymphopenic hosts after conversion into Th1 cells. Eur J Immunol 39: 216–224. 19130584 10.1002/eji.200838475 PMC2755057

[31] Han G, Wang R, Chen G, Wang J, Xu R, et al (2010) Interleukin-17-producing gammadelta+ T cells protect NOD mice from type 1 diabetes through a mechanism involving transforming growth factor-beta. Immunology 129: 197–206. 19824917 10.1111/j.1365-2567.2009.03166.x PMC2814462

[32] Markle JG, Mortin-Toth S, Wong AS, Geng L, Hayday A, et al (2013) gammadelta T cells are essential effectors of type 1 diabetes in the nonobese diabetic mouse model. J Immunol 190: 5392–5401. 23626013 10.4049/jimmunol.1203502 PMC3836168

[33] Arif S, Moore F, Marks K, Bouckenooghe T, Dayan CM, et al (2011) Peripheral and islet interleukin-17 pathway activation characterizes human autoimmune diabetes and promotes cytokine-mediated beta-cell death. Diabetes 60: 2112–2119. 21659501 10.2337/db10-1643 PMC3142078

[34] Honkanen J, Nieminen JK, Gao R, Luopajarvi K, Salo HM, et al (2010) IL-17 immunity in human type 1 diabetes. J Immunol 185: 1959–1967. 20592279 10.4049/jimmunol.1000788

[35] Marwaha AK, Crome SQ, Panagiotopoulos C, Berg KB, Qin H, et al (2010) Cutting edge: Increased IL-17-secreting T cells in children with new-onset type 1 diabetes. J Immunol 185: 3814–3818. 20810982 10.4049/jimmunol.1001860

[36] Kuriya G, Uchida T, Akazawa S, Kobayashi M, Nakamura K, et al (2013) Double deficiency in IL-17 and IFN-gamma signalling significantly suppresses the development of diabetes in the NOD mouse. Diabetologia 56: 1773–1780. 23699989 10.1007/s00125-013-2935-8

