# Supplementary figures and images for: Potential Role of IL-17-Producing iNKT Cells in Type 1 Diabetes
Source: PLoS One. 2014 Apr 30;9(4):e96151. doi: 10.1371/journal.pone.0096151 (PMC4005752; doi:10.1371/journal.pone.0096151)

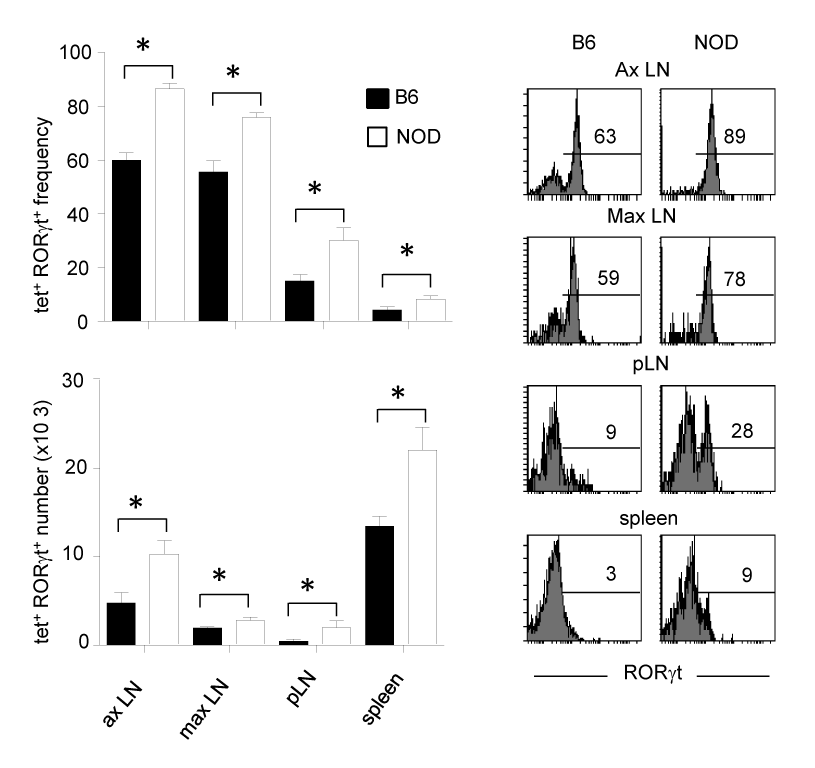

Supplement: Figure S1 — RORγt+ iNKT cells are increased in NOD mice compared with C57BL/6 mice. Axillary (ax), maxillary (max), and pancreatic (p) LNs, and spleen cells from 12-wk-old C57BL/6 or NOD female mice were stained with CD1d-tetramers (tet) and antibodies directed against the transcription factor RORγt. The frequency and the absolute number of RORγt+ cells among tet+ cells (left), and representative histogram plots (right) are shown. Numbers represent percentages. Data are presented as mean ± SD and are from 5 experiments where 3 to 4 mice per group were used in each experiment. *p<0.05, using non-parametric Mann-Whitney U test to determine significance. (TIF) [file pone.0096151.s001.tif]

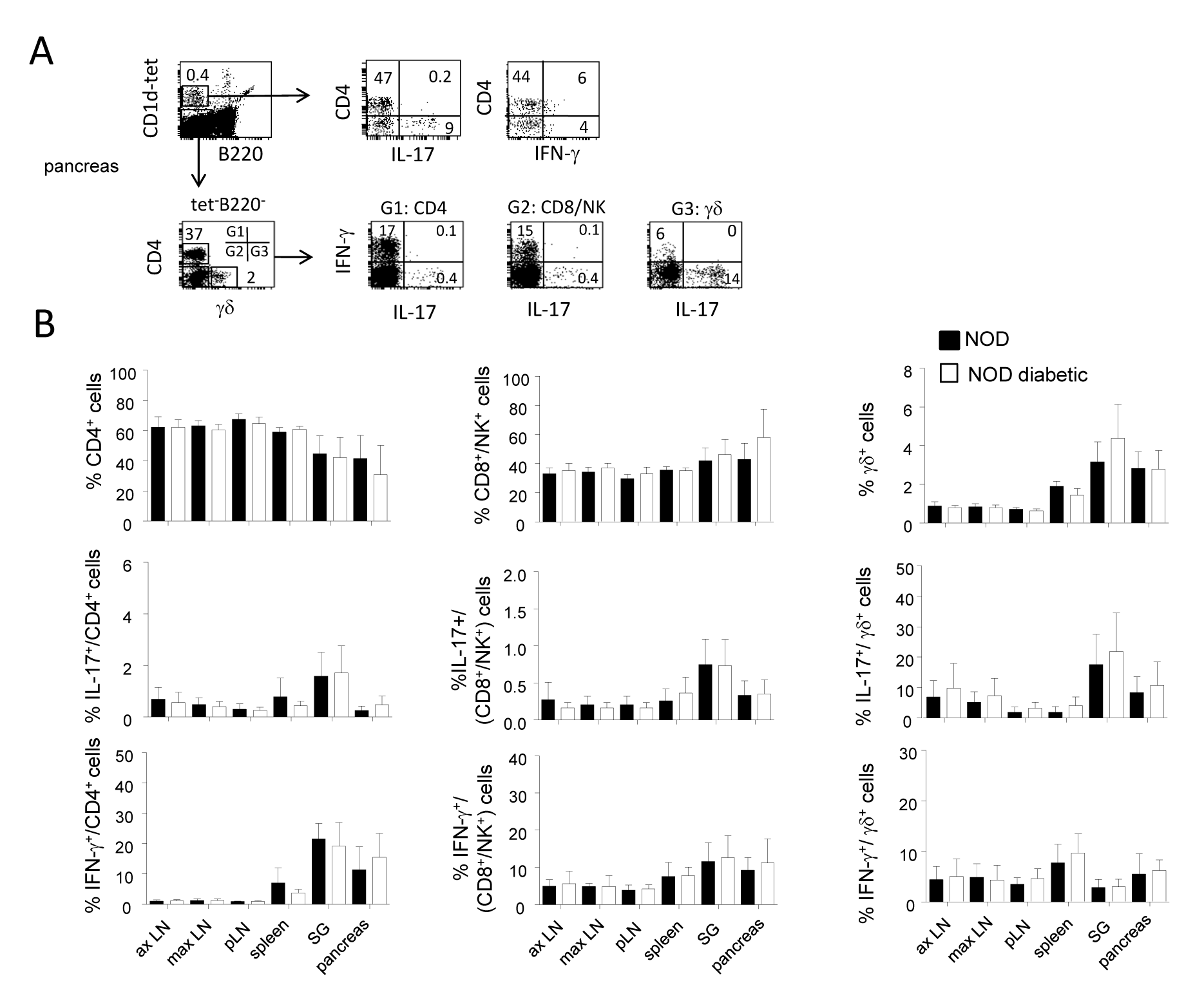

Supplement: Figure S2 — Unaltered cytokine production of CD4+, NK+/CD8+, and γδ+ cells of diabetic NOD mice. A, Axillary (ax), maxillary (max), and pancreatic (p) LNs, and spleen cells from same aged diabetic or control non diabetic NOD female mice (20- to 22-wk-old) were stimulated with PMA/Ionomycin in the presence of BFA for 4 hours. Cells were then surface stained with CD1d-tetramers and antibodies directed against B220, CD4, and TCRγδ, followed by intracellular staining to detect IL-17A (IL-17) and IFN-γ. Shown are representative dot plots and gating strategies to determine cell population frequencies and cytokine production. Numbers represent percentages. B, Shown is the frequency of CD4+, CD8+/NK+, and γδ+ cells and the frequency of IL-17+ and IFN-γ+ cells among these cells. Data are presented as mean ± SD and are from 4 experiments where 4 to 5 mice per group were used in each experiment. (TIF) [file pone.0096151.s002.tif]

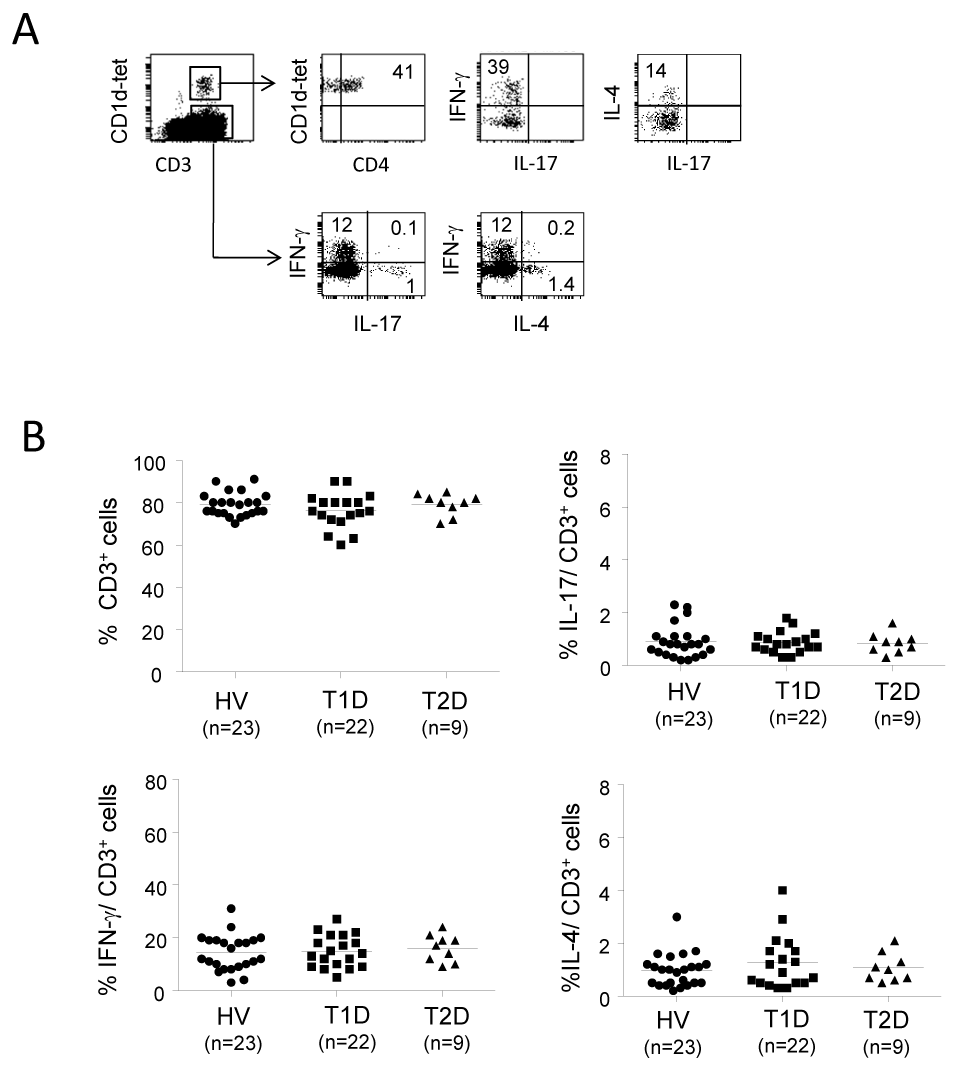

Supplement: Figure S3 — Unaltered cytokine production of CD3+ cells in patients with type 1 diabetes. A, Fresh human PBMCs were stained with CD1d-tetramers (tet) and anti-CD3 antibodies after being stimulated with PMA/Ionomycin. Cells were then subjected to intracellular staining to assess IL-17, IFN-γ, or IL-4 production. Shown are representative dot plots of tet-staining and IL-17, IFN-γ, and IL-4 production among tet+ or CD3+tet− cells from a patient with type 1 diabetes (T1D). B, Shown is the frequency of IL-17+, IFN-γ+, and IL-4+ cells among CD3+tet− cells in Healthy volunteers (HV), T1D patients, and patients with type 2 diabetes (T2D). Each symbol represents one individual and horizontal bars indicate mean ± SD. n: number of subjects tested. (TIF) [file pone.0096151.s003.tif]

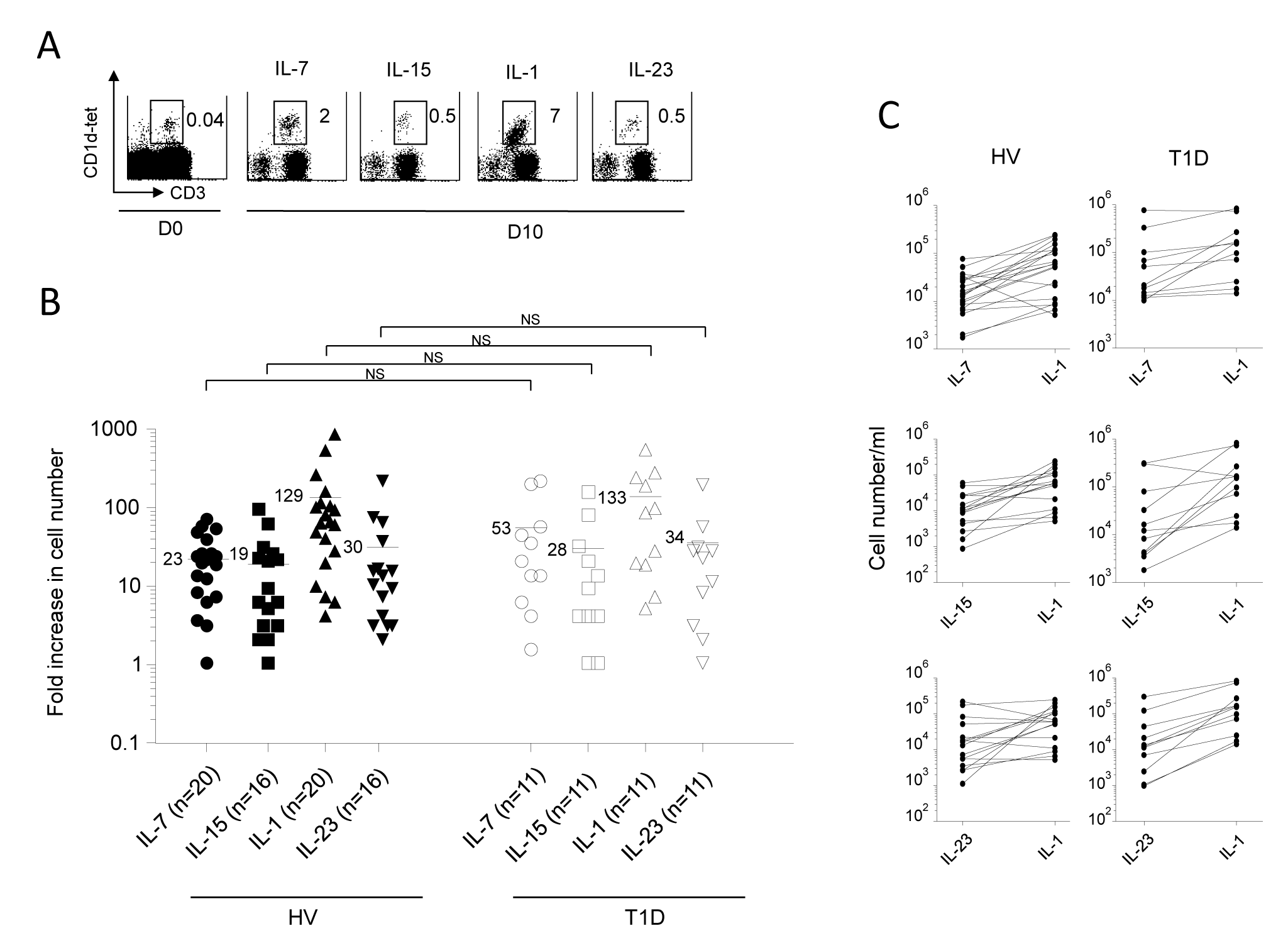

Supplement: Figure S4 — iNKT cells expand in vitro in the presence of the proinflammatory cytokine IL-1β. A, Human PBMCs from healthy volunteers (HV) or type 1 diabetes (T1D) were cultured for 10 days in the presence of IL-2, α-GalCer and the mentioned cytokine. Proliferation of iNKT cells (gated as tet+CD3+ cells) is evaluated. Representative dot plots of CD1d tetramer staining from a T1D patient before (D0) and after expansion (D10) under the mentioned conditions are shown. Numbers represent percentages. B, Fold increase of iNKT cell number for each subject tested (after 10 days of culture/at the start of the culture) is shown. Each symbol represents one individual and horizontal bars with numbers indicate mean. NS: not significant. n: number of subjects tested. C, Shown are paired comparison between tet+ cell numbers observed after 10 days of culture with the mentioned cytokines. (TIF) [file pone.0096151.s004.tif]
